# Experiences of Wearable Technology by Persons with Knee Osteoarthritis Participating in a Physical Activity Counseling Intervention: Qualitative Study Using a Relational Ethics Lens

**DOI:** 10.2196/30332

**Published:** 2021-11-12

**Authors:** Jenny Leese, Graham MacDonald, Catherine L Backman, Anne Townsend, Laura Nimmon, Linda C Li

**Affiliations:** 1 Faculty of Medicine The University of British Columbia Vancouver, BC Canada; 2 Arthritis Research Canada Vancouver, BC Canada; 3 Ottawa Hospital Research Institute Ottawa, ON Canada; 4 Faculty of Medicine University of Ottawa Ottawa, ON Canada; 5 Faculty of Health and Medicine Lancaster University Lancaster United Kingdom

**Keywords:** relational ethics, physical activity, wearable, arthritis, qualitative

## Abstract

**Background:**

Current evidence indicates physical activity wearables could support persons with knee osteoarthritis (OA) to be more physically active. However, recent evidence also identifies some persons with arthritis experience guilt or worry while using a wearable if they are not as active as they feel they should be. Questions remain around how persons with knee OA experience benefits or downsides using a wearable in their everyday lives. Better understanding is needed if wearables are to be incorporated in arthritis self-management in ethically aware ways.

**Objective:**

Using an ethics lens, we aimed to describe a range of experiences from persons with knee OA who used a wearable during a physical activity counseling intervention study.

**Methods:**

This is a secondary analysis of qualitative interviews nested within a randomized controlled trial. Guided by phenomenography, we explored the experiences of persons with knee OA following participation in a physical activity counseling intervention that involved using a Fitbit Flex and biweekly phone calls with a study physiotherapist (PT) in an 8-week period. Benefits or downsides experienced in participants’ relationships with themselves or the study PT when using the wearable were identified using a relational ethics lens.

**Results:**

Interviews with 21 participants (12 females and 9 males) aged 40 to 82 years were analyzed. Education levels ranged from high school graduates (4/21, 19%) to bachelor’s degrees or above (11/21, 52%). We identified 3 categories of description: (1) participants experienced their wearable as a motivating or nagging influence to be more active, depending on how freely they were able to make autonomous choices about physical activity in their everyday lives; (2) some participants felt a sense of accomplishment from seeing progress in their wearable data, which fueled their motivation; (3) for some participants, sharing wearable data helped to build mutual trust in their relationship with the study PT. However, they also expressed there was potential for sharing wearable data to undermine this trust, particularly if this data was inaccurate.

**Conclusions:**

Findings provide an early glimpse into positive and negative emotional impacts of using a wearable that can be experienced by participants with knee OA when participating in a randomized controlled trial to support physical activity. To our knowledge, this is the first qualitative study that uses a relational ethics lens to explore how persons with arthritis experienced changes in their relationship with a health professional when using a wearable during research participation.

## Introduction

Affecting an estimated 302 million people worldwide, osteoarthritis (OA) is one of the most common forms of arthritis, and it is a leading cause of disability among older adults [1-5]. Evidence-based practice guidelines recommend physical activity as a key component of optimal OA self-management due to its beneficial effects on pain, mobility, and quality of life [6-8]. However, most people with OA do not meet these recommendations, and supporting persons with knee OA to be more active remains problematic [9,10].

The use of consumer-available, activity-monitoring wearable devices offers a promising strategy to increase physical activity among persons with knee OA. Indeed, literature exists to indicate that wearable technology-enabled interventions can significantly increase moderate-to-vigorous physical activity (MVPA) among adults with knee OA [11-15]. These findings build on previous research to suggest that these interventions may significantly improve MVPA participation if the devices are integrated as part of a multifaceted intervention involving counseling with a health professional [16,17].

Evidence exists, however, to indicate that using digital health technologies (including wearables) in the practice of self-monitoring and self-management may be experienced positively or negatively by persons with chronic illness in the context of their everyday lives [18,19]. In a recent synthesis of qualitative evidence, Leese et al [20] provided an early glimpse of ethical issues identified in the perspectives of persons with arthritis on the use of wearables to support physical activity participation in their everyday lives. These ethical issues were expressed by persons with arthritis as benefits and downsides in their relationships with themselves (ie, their self-perception) and their health professionals. It was found, for example, that persons with OA expressed a general opinion that communication with their health professionals could be enhanced or challenged through the use of a physical activity wearable in their everyday self-management, depending on the quality of their existing relationship [21-23]. However, empirical evidence on the experiences of persons with OA participating in physical activity interventions involving a health professional and using a wearable device is minimal [20]. Furthermore, there is little knowledge on the impact of using a wearable in patient-health professional relations, which are laden with power dynamics [24]. Leese et al [20] also identified that while some persons with chronic illness, including OA, spent more time in physical activity and felt more confident about managing their health while using a wearable, others experienced guilt or worry when they were not as active as they felt they should be while using wearables [25].

An in-depth understanding of a fuller spectrum of experiences of persons with OA is needed if wearable-enabled physical activity counseling programs to support arthritis self-management are to be implemented in ways that appropriately consider ethical issues, such as benefits and downsides encountered by patients. Thus, this study aimed to examine a range of experiences encountered by persons with OA who participated in a study of a wearable-enabled physical activity counseling intervention, with particular attention paid to any influences on participants’ relationships with themselves and with the physiotherapist (PT).

## Methods

### Study Design

This study was a qualitative secondary analysis of semistructured interview data with participants of a mixed-methods study named MONITOR-OA [11]. The original study involved a 6-month proof-of-concept randomized controlled trial (RCT) to examine the efficacy of a technology-enabled counseling intervention for increasing MVPA among persons with knee OA and a qualitative component to describe the participants’ experiences of the intervention [11]. Fundamental qualitative description and conventional content analysis methods guided the qualitative component of the original study [26,27]. As a complete data set was already present, our secondary analysis used phenomenographic analytical methods and introduced a relational ethics lens to shape interpretations of the original interview data, in line with Varpio et al’s [28] description of a theory-informing inductive data analysis study design [29,30]. Relational ethics is a broad theoretical lens that continues to evolve from critiques of a strong individualistic perspective that dominated traditional bioethics discourse [31-34]. These critiques highlight the complex ways in which persons develop within (and are inherently shaped by) relationships (personal and institutional, past and present) that are an integral part of one’s life. They expand on traditional bioethics principles by locating ethical issues in the context of everyday relational settings [31]. Phenomenography is concerned with relations between a person and a specified aspect of the world as it appears to them [29]. Phenomenographic analytic methods and a relational ethics lens thus offer appropriate theoretical grounding to explore the particular focus of our study on everyday ethical issues experienced by participants in their relationships with themselves and the PT while using a physical activity wearable. Our approach rests on assumptions that reality is socially and experientially constructed, and to understand these realities, researchers need to explore the meanings constructed by individuals or groups.

The RCT component of the original study took place between November 2015 and June 2017 in Vancouver, Canada. Participants attended a 1.5-hour session, where they received (1) 15-minute group education about physical activity, (2) a Fitbit Flex (Google LLC), and (3) individual counseling with a study PT who was trained in motivational interviewing [35]. The individual counseling session followed the brief action planning approach, whereby PTs guided participants to identify activity goals, develop an action plan, and identify barriers and solutions [36].

Participants were asked to wear the Fitbit wristband 24 hours a day except during water-based activities or when recharging the battery. The physical activity data were wirelessly synchronized with Fitbit’s online Dashboard that could be viewed only by the participants and their study PT. During the intervention period, the PT reviewed the participants’ physical activity data on the Dashboard and reviewed their activity goals during 4 biweekly phone calls. Participants could also contact the PT via email in-between the scheduled calls. After the 8-week intervention concluded, all participants were invited to take part in an interview about their experiences.

The research protocol was approved by the University of British Columbia Behavioral Research Ethics Board (H14-01762) and was published in clinicaltrials.gov (NCT02315664). Informed consent to use interview data for the secondary analysis was obtained from all participants at the time of the original study.

### Participants

Further details of the original RCT have been described elsewhere [11]. Briefly, individuals were eligible if they were adults living in Vancouver, Canada, with a physician-confirmed diagnosis of knee OA or who passed two criteria for early OA: (1) aged 50 years or older and (2) experienced knee pain during the previous year lasting more than 28 separate or consecutive days [37]. Individuals were excluded if they (1) had been diagnosed with inflammatory arthritis, connective tissue diseases, fibromyalgia, or gout; (2) used antirheumatic drugs or gout medications; (3) had previously undergone knee arthroplasty; (4) had suffered an acute knee injury in the past 6 months; (5) had a body mass index of 40kg/m^2^ or higher; (6) had received a steroid injection or a hyaluronate injection in the last 6 months; and (7) were using medications which impaired physical activity tolerance (eg, beta-blockers), or had an inappropriate level of risk for increasing their physical activity. Participants were also excluded if they did not have access to a computer in their home or did not have a personal email address.

The original RCT had 61 participants, of which 56 completed the in-depth interview after the intervention. As this study aimed to examine the range of experiences among participants, sampling was mostly theoretical to maximize variation across demographic characteristics (eg, age, sex, and education). Our analysis focused on a purposive subsample of 21 of these interview participants (13 females and 8 males), ranging in age from 40 to 82 years (Table 1; participants chose their pseudonyms). Participants came from a variety of household compositions, with education levels ranging from high school graduate (4/21, 19%) to a bachelor’s degree or above (11/21, 52%), and annual household incomes ranging from under CAD $12,000 (US $9,750; 1/21, 5%) to over CAD $100,000 (US $81,240; 4/21, 19%).

**Table 1:** Participants’ sociodemographic characteristics.

Participant pseudonym	Age at consent	Sex	Education	Annual household income, CAD^a^	Marital status	Living status	Other conditions
Martha	82	Female	Bachelor’s degree or higher	$60,001-$80,000	Widowed	Alone	Circulation problems; cancer
Lenny	68	Female	Trades certificate, vocational school diploma, apprenticeship	$40,001-$60,000	Widowed	Alone	None reported
Anne	61	Female	Bachelor’s degree or higher	$60,001-$80,000	Separated/divorced	Alone	Allergies; breathing problems; osteoporosis; osteopenia
Marco	64	Male	Bachelor’s degree or higher	Over $100,000	Married/common law	Significant other	Allergies
Don	63	Male	Bachelor’s degree or higher	Prefer not to answer	Married/common law	Significant other	High blood pressure; allergies
Bruce	58	Male	Grade 11 to 13 (including GED^b^)	Over $100,000	Married/common law	Significant other	High blood pressure
Darius	64	Male	Nonuniversity certificate below Bachelor’s level	$60,001-$80,000	Married/common law	Significant other	Allergies; kidney, bladder, or urinary problems
Minnekhada	58	Male	Grade 11 to 13	$60,001-$80,000	Married/common law	Significant other	None reported
Gavin	61	Male	Bachelor’s degree or above	Over $100,000	Married/common law	Significant other	None reported
Joe	71	Male	Bachelor’s degree or above	$24,001-$40,000	Never married	Alone	None reported
Hazel	63	Female	Bachelor’s degree or above	$60,001-$80,000	Separated/divorced	With relatives or others	Digestive system problems; allergies; breathing problems; psoriasis; mental health or emotional problems
Tony	77	Male	Trades certificate, vocational school diploma, apprenticeship	$12,001-$24,000	Married/common law	Significant other	Cancer
Yoda	40	Female	Bachelor’s degree or above	Prefer not to answer	Married/common law	With children	None reported
Zed	56	Female	Nonuniversity certificate below Bachelor’s level	Prefer not to answer	Separated/Divorced	With children	Allergies; diabetes; breathing problems
Denny	61	Female	Non-university certificate below Bachelor’s level	$80,001-$100,000	Married/common law	With children	None reported
Logan Kale	41	Female	Grade 11 to 13	$24,001-$40,000	Married/common law	With children	Fibromyalgia
Olivia	69	Female	Bachelor’s degree or above	$12,001-$24,000	Separated/divorced	Alone	Diabetes; cancer
Jane	53	Female	Grade 11 to 13	Prefer not to answer	Separated/divorced	With relatives or others	Cerebrovascular problems; headaches
Daenerys	57	Female	Bachelor’s degree or above	Over $100,000	Married/common law	Alone	Digestive system problems; allergies; kidney, bladder, or urinary problems
Sansa	57	Female	Bachelor’s degree or above	$80,001-$100,000	Married/common law	With children	None reported
Biker	61	Female	Non-university certificate below Bachelor’s level	$60,001-$80,000	Married/common law	Significant other	Skin problems

^a^A currency exchange rate of CAD $1=US $0.80 is applicable.

^b^GED: general education diploma.

### Interviews

The first author (JL) conducted 13 of the 21 in-depth one-on-one interviews, and the second author (GM) conducted the remaining 8 interviews. JL (cisgender woman) and GM (cisgender man) were PhD Candidates with approximately 10 years of experience each in qualitative interviewing. All interviews followed a semistructured interview guide including the following questions: Can you tell me about any physical activity you do? Since taking part in this study, have you noticed any changes in your physical activity? How easy or difficult has it been using the Fitbit? What about your experience with the study PT? Prompts and probes were also used as reminders during active listening, and considerable attention was given to the interviewee articulating their experience in their own words. Interviews (lasting approximately 60-90 mins) were audio-recorded and transcribed verbatim.

### Data Analysis

Phenomenographic analytical methods were used to describe the different (positive and negative) ways in which persons with knee OA experienced the physical activity counseling intervention and the various meanings they attributed to this phenomenon in their world [29]. Although the roots of phenomenographic research methods lie in learning studies, the methods have been applied to a variety of other issues inside and outside the field of education [38-41]. We use this analytic approach for two reasons. First, the MONITOR-OA RCT provided a complete data set of interview transcripts in which participants’ experiences using a wearable in a physical activity intervention was a prominent issue. Second, this study focused on exploring differences among the collective experience that persons with OA had when participating in the intervention. Both reasons aligned with phenomenography, justifying it as an appropriate approach for analysis.

We analyzed the data through a relational ethics lens, following Heaton’s [42,43] definition of supra-analysis, which is one type of secondary analysis involving examining pre-existing data from a new theoretical perspective, thereby transcending the aims and focus of the original research. We reused our self-collected data to examine it from a new theoretical perspective than the primary study, specifically a relational ethics perspective. Relational ethics places the principles of bioethics that are traditionally used to guide moral practice in health care (eg, autonomy, nonmaleficence, beneficence, and justice) within the context of close-up relationships [30,34,44]. It assumes that all relationships are moral and attends to the commitment to ethical action in one’s relationship to oneself and the other in every situation or encounter on an ordinary everyday basis [34,44]. Issues of relational autonomy, such as engagement and partnership, have been previously identified in perspectives of persons with arthritis on their use of wearable devices to self-monitor physical activity [20]. Building on this earlier research, our study focused on participants’ experiences of the benefits or drawbacks of using a wearable, with a particular interest in any impacts experienced in their relationships with themselves and with the study PT. Therefore, relational ethics is a suitable conceptual lens through which to continue the exploration of ethical issues identified from the perspectives of persons with arthritis.

Our analysis was informed by the 7 steps of phenomenographic analysis as described by Sjöström and Dahlgren [41]: familiarization, compilation, condensation, grouping, comparison, naming the categories, and contrastive comparison (Textbox 1). Data were reviewed carefully for themes that attend to morality in everyday relationships, including (but not limited to) self-control, engagement, and partnership [30,34,44]. Regular meetings were held with the research team, some of whom had experience in phenomenographic analysis, to enhance rigor and check, test, and probe preliminary findings [45].

Informed by calls to critically consider the relevance of notions of saturation within our study’s context, we did not consider saturation to be meaningful to our research objective and methodological orientation because we did not seek to saturate theoretical categories, themes, or data [46-49]. Our sampling and analysis ceased when the research team reached an agreement that findings were sufficiently varied to describe a range of participants’ experiences relevant to our research objective. Based on the previous experience of phenomenographers, 15 to 25 interviews are typically preferred in a phenomenographical study [50]. While we acknowledge that what is determined as an appropriate sample size for one qualitative study is not necessarily an appropriate sample size for another qualitative study [51], the combination of team agreement regarding content and the number of interviews compared to past phenomenographic studies supported our decision.

**Data analysis process.**

**Familiarization:** Our analytic method began with JL reading and re-reading all of the transcripts. GM also read and re-read a varied sample of 4 of these transcripts.

**Compilation:** Next, JL and GM independently identified excerpts of data in these 4 transcripts that were found to be of interest for the question being investigated.

**Condensation:** JL and GM met regularly to discuss and narrow down a selection of data excerpts from the 4 transcripts that they identified as relevant.

**Grouping:** JL and GM sorted quotes within this selected data pool into piles based on similarity to create a preliminary set of categories of description. At the end of this phase, the following preliminary categories were identified: feeling accountable, changing awareness/perceptions, increasing physical activity, feeling better, having an objective measure, participant-physio relationship, reaching physical activity goals, relating to the Fitbit, emotional impacts of using Fitbit, and making progress.

**Comparison:** Next, using QSR International NVivo software (version 12) to organize data, JL tested the preliminary categories against the remaining transcripts. She sorted and re-sorted data extracts from the remaining transcripts. This process entailed refining or collapsing the preliminary categories. Preliminary categories were also compared and differentiated from one another in terms of differences (making criterion attributes for each category explicit). All team members met to discuss the categories as they were developed.

**Naming:** 3 final categories were defined to emphasize their essence were agreed upon through discussion with all team members.

**Contrastive Comparison:** Informed by a discussion with all team members, JL described the unique character of each final category as well as the resemblances between them. She also checked the final categories against the original transcript data.

## Results

### Overview

Phenomenographic analysis provided a rich interpretation of how persons with knee OA experienced benefits and drawbacks in using a Fitbit during their participation in a physical activity counseling intervention study. Our analysis revealed 3 main categories: (1) making choices about physical activity with or without a wearable, (2) emotional dimensions of adding awareness about physical activity, and (3) reviewing wearable data with the study PT: issues of accountability and trust. Key quotes are presented in Textboxes 2-4 to illustrate each category, and supplementary supporting quotes can be found in Multimedia Appendix 1.

### Making Choices About Physical Activity With or Without a Wearable

Participants described how their Fitbit had influenced their choices to increase their walking to reach their daily step goal (Textbox 2, quotes 1-4). Some attributed human-like traits to their Fitbit when describing its influence on their activity choices (Textbox 2, quotes 1-3). For example, Hazel described her Fitbit as “a little person on my wrist…a little friend” that was a welcome source of friendly and gentle encouragement to meet her step goals. For her, Fitbit was “happy when I do 10,000 steps…just like a friend supporting me…There’s a gentle persuasion.” Others expressed how they felt pressured by their Fitbit to meet their activity goals. Martha, for example, felt ambivalently toward her Fitbit’s influence in her decision to walk more, reflecting that “it probably gives me some incentive to walk a little further just to placate the Fitbit…it nags you…I’m fine with that…it’s probably a good thing to have something that makes you get up and go.” Denny felt “forced to kind of do some more activities” by her Fitbit in the evenings when she felt too tired. Sansa indicated she felt a sense of responsibility to meet her activity goals and viewed the Fitbit as “there to keep me accountable.”

Some participants described certain days they did not engage with their Fitbit; for example, on days they were experiencing “too much pain” or on “some days I don’t care” to be more active. Zed found that Fitbit’s influence did “start me going” to meet step goals “in the beginning” of the study, and its influence “wanes over time” once his increase in walking became part of his habitual routine. Others explained that wearing their Fitbit did not add any value as they were making choices to meet their activity goals regardless (Textbox 2, quote 5). As a busy mother, Yoda found that Fitbit was generally irrelevant as meeting her activity goals was not her main priority (something for which she suggested “shows perhaps an inherent bad attitude”). She recounted, “I wasn’t invested that I absolutely had to do, come hell or high water, these steps so I’d be like yeah, I just didn’t walk very much today.”

**Supporting quotes for “Making choices about physical activity with or without a wearable.”**

**1_Hazel:** the Fitbit…It’s like a little person on my wrist…it’s a little friend…and I tap into it and go, “I’m almost to my 10,000 but I could do a bit more”…throughout my day I can refer to it and I think it’s on my side. It’s happy when I do 10,000 steps…just like a friend supporting me, encouraging me…whether I achieve it or not it’s still there with me…Whether I do it or not, it’s up to me. There’s a gentle persuasion…a Fitbit helps me feel less alone…it’s huge for me because you know most of my life I’ve not had a lot of support and so support is huge, just huge.

**2_Sansa:** when I go on there and I see that I haven't reached my six or whatever, I'll in the evening go for you know a five-minute walk or a 10-minute walk or you know try to do that after dinner. So it does give me a little bit more kind of accountability or kind of check where I'm at. I think it's kind of like I have a partner in crime, it's just kind of there to keep me accountable.

**3_Martha:** It probably gives me some incentive to walk a little further just to placate the Fitbit…I forget to check but I think it does give you an incentive to get out and do something because it’s there and it nags you…I’m fine with that…it’s probably a good thing to have something that makes you get up and go.

**4_Denny:** I finally get home say around seven in the evening…just kind of want to eat and then just do nothing…I know you’re supposed to move [laughs]…But sometimes I’m just too tired and in the evenings, I’m forced to kind of do some more activities…having the Fitbit it does make me feel I need to move more…I have definitely gone for more walks.

**5_Joe**: I max out in my activities, so I don’t need this monitoring as a way of positive feedback or gratification to give me incentive. I personally don’t need that…I’m actually walking and all of that. If somebody says, “Oh you should dance more in the evening,” I say, “Well no I can’t dance anymore.” I can only go so far then I drop dead right? So I’m maxed out. I can’t add much more here…So when this six months is over, I’ll take the Fitbit and throw it away because it has no relevance to my life…I know what I’m doing and I don’t care what this little machine tells me.

### Emotional Dimensions of Adding Awareness About Physical Activity

Some participants highlighted there was an emotional dimension to the heightened awareness of their physical activity levels they experienced through using the wearable (Textbox 3, quotes 1-3). Some described how this added awareness about their activity levels prompted feelings of accomplishment and gratification, which fueled their motivation. For example, Daenerys recalled, “It lets me feel as though I’m accomplishing something every day…I feel pretty happy…and then it’s kind of fun to see how much more I can do.” Olivia also felt “instant gratification” as she could see improvement in Fitbit’s feedback on her step goals.

While Logan Kale felt “happiness, accomplishment” when she reached her activity goal most days, she also found that on days when she was “in too much pain” to reach her step goal, she would “try not to beat myself up over it” and “kind of get stuck on that hamster wheel of negative thoughts and have to zip it.” Biker commented that she did not “feel bad” when Fitbit data showed she had not met their step goal because “I like to exercise and so being active is not an issue for me…actually I kind of find it a kind of cozy feeling thinking ‘I haven’t completed everything. There’s still more to do.’” She expected, however, that others who were less active than her “might feel sad” if they did not meet a physical activity goal.

**Supporting quotes for “Emotional dimensions of adding awareness about physical activity.”**

**1_Daenerys:** I will always keep the Fitbit and always have one I think because it lets me feel as though I’m accomplishing something every day, like I have it set to a pretty low number of steps every day. It’s set to 3000 but when I look at my results I can be over 3000. When it goes off during the day, I feel pretty happy about, “Okay, I’ve accomplished that much today,” and then it’s kind of fun to see how much more I can do.

**2_Logan Kale:** when I would be close to my steps, if I would see…you just tap it [the Fitbit] and then you’ll know if you’re close or not and I would just make that extra effort to meet that mark. Like instead of driving to work, I would walk to work. On most days it was easy…just seeing that number of how you’re so close. “Got to get over that hump.” Once a goal you’ve set and, you know, when you reach that goal, you feel good about it. It’s just happiness, accomplishment. The other days I’m just in too much pain. I’m like, “I’m not walking home.” I want to be that person going on those hikes. I don’t want to be that person just sitting there. You want to always try to do better the next week but then if it doesn’t happen, I try not to beat myself up over it anymore because, you know, the next day could be better. I’m like, “Okay well you didn’t do well this week. What’s the problem? You shouldn’t be doing that. You shouldn’t be doing this. You should be doing that,” and I just kind of get stuck on that hamster wheel of negative thoughts and have to zip it.

**3_Biker:** [responding to *you mentioned that there were some goals that you didn’t meet…*] I don’t feel bad at all. [Laughs] I just kind of go, “Oh that’s life” because I know that I’m keeping really active so yeah it’s not a problem to me. I guess if I thought, “Gee I’m not very active and I’m not meeting any of my goals” then I might feel sad about it but because I know that I like to exercise and so being active is not an issue for me. So the goals I set are kind of like…in a perfect world this is what I would like to do but the world isn’t perfect and it’s okay. You know, I’m working, I’m certainly getting tons of weekly exercise in and so if I don’t meet some aspect of it, it’ll be okay. You know also I guess I can also look at it and kind of go, “If I really, really wanted to do Yoga, I could put a DVD on and do some at home” but again, I like the Yoga for the social aspect so that’s not much of an incentive to do it on my own in my living room…actually I kind of find it a kind of cozy feeling thinking, “I haven’t completed everything. There’s still more to do.” So I’m not taking it kind of like I’m a failure, I’m taking it more as, “Oh there’s still more to do and you can keep on growing, keep on improving” so.

### Reviewing Wearable Data With the Study PT: Issues of Accountability and Trust

Participants described how their choices to be physically active had been influenced because the study PT had access to review wearable data on their physical activity (Textbox 4, quotes 1-2). For example, Denny described how she had “tried a little bit harder [laughs] maybe for a few weeks” to reach her activity goals because she knew the study PT had access to her wearable data and found this to be “very, very encouraging.” Darius also indicated that he felt a sense of accountability to meet the physical activity goals he had agreed with the study PT, as he recalled “a little bit of pressure…I need to watch to keep my promise.”

For one participant, accountability issues intertwined with trust issues in his relationship with the study PT when reviewing his wearable data together (Textbox 4, quotes 3-4). Recounting how his wearable data had not always accurately tracked his step count because it sometimes had “slipped into sleep mode,” Gavin described a phone call he had with the study PT in which he explained that his subjective account of his physical activity should be trusted as more credible than his wearable data, and he should not be held accountable, because “it wasn’t my fault I didn’t make my 10,000.” When he felt his Fitbit had tracked his step count more accurately, Gavin described this data as “some form of proof” that could be shared with the study PT “for her to gauge” his activity. He also commented that “if [Fitbit data] wasn’t there… then ‘oh yeah I climbed Mt. Everest this weekend’…I could’ve been making up anything about that,” emphasizing that, when accurate, Fitbit data may give a more reliable account of his physical activity than himself.

**Supporting quotes for “Reviewing wearable data with the study PT: Issues of accountability and trust.”**

**1_Denny:**…you have a knowledgeable person telling you, you’re doing the right thing type thing…I tried a little bit harder [laughs] maybe for a few weeks…because you actually have another person kind of monitoring you and you also…you want to try…it was very, very encouraging…I think it’s really good for her to be able to see and for me to know that somebody is monitoring me. I think maybe that makes me [laughs] take a few more steps maybe.

**2_Darius:** It’s just a little bit of pressure of keeping the, the steps they...Because I, I need to watch to keep my promise, you know, as far as I can…she [the physiotherapist] called me like every two weeks. I think her purpose is to motivate me for keeping my, my promise to keep activities.

**3_Gavin:** I do have a grump with the Fitbit over the times where it’s gone into sleep mode so many times…Before I know it I’ve lost 3000 steps…you go like, “I get a lot more steps today than that’s showing me. I know it’s slipped into the sleep mode activity and it’s not”…I figured I should have my entire European boot badge by now so it’s not fair…I knew I was getting a phone call from the physio and I said, “Oh yeah but it wasn’t my fault I didn’t make my 10,000. This stupid band didn’t log on properly.” Oh she said, “Oh yeah, it happens.”

**4_Gavin:** I think [Fitbit data] at least gave a better conversational point in terms of seeing how you were doing, a reference check for the physio when we’re checking in. If it wasn’t there it would really…there would be nothing for her to gauge because then, “What did you do?” “Oh yeah I climbed Mt. Everest this weekend and am feeling really good. Kilimanjaro is tomorrow. Not bad.”… we would’ve been talking in a fairy land because I could’ve been making up anything about that…”

## Discussion

### Principal Findings

Our findings provide novel insight into different ways in which persons with knee OA experienced their use of a wearable positively or negatively during their research participation. Firstly, the contradictions in the data were fascinating. Participants experienced their wearable positively as a motivating influence and more negatively as a nagging reminder to be more active. From a relational ethics perspective, these findings shed light on how participants’ experience of their wearable impacts their autonomy positively or negatively.

Autonomy is a central notion in modern health care ethics that is understood as the capacity to direct one’s own choices freely and intentionally [31]. A relational approach builds on this traditional understanding of autonomy by focusing attention on relationships and interdependencies that may support or impair a person’s capacity for autonomy [52,53]. The relational autonomy perspective highlighted that many participants experienced the relationship with their wearable as a support to their autonomy. The device helped them take more control in making choices to be more active. However, some participants who described their capacity to make autonomous choices about their physical activity as impaired (eg, due to tiredness) experienced tension and ambivalence in the relationship with their wearable, as they described feeling pressured or forced to be more active at times when they did not entirely wish to move. These findings align well with previous research suggesting that using a wearable may be experienced as autonomy-enhancing or autonomy-undermining by persons with arthritis, regardless of whether they used the wearable to support physical activity during research participation or as part of their everyday self-management [20]. Leese et al [23] have reported some persons with arthritis experienced their wearable as a motivating or autonomy-enhancing support in their everyday self-management when used as a “nice reinforcement” to an already physically active lifestyle.

Findings in our study also indicated positive and negative experiences of participants using a wearable, depending on how freely they were able to direct their own choices to be active given their specific situation or set of circumstances. They resonate with a relational ethics approach that recognizes exercising autonomy requires relevant capabilities, which are dynamically shaped by a person’s situation or set of circumstances [54]. They also resonate with calls from some health professionals for academic literature to facilitate the positive development of self-tracking technology in self-management by reflecting on context-relativity (rather than focusing on “ideal” situations) [55]. We posit that further research is thus warranted to build a greater understanding of the everyday contexts in which the use of a wearable may be experienced as autonomy-enhancing or autonomy-undermining by persons self-managing chronic illness.

At this early stage of the potential integration of wearables into arthritis self-management, our findings can contribute to ongoing conversations in clinical practice. They suggest health professionals may wish to carefully consider a person’s capability to make autonomous choices about their physical activity if using a wearable in their everyday self-management. They, therefore, align well with research suggesting how health professionals may tailor their support for arthritis self-management in ways that take a person’s capacities to engage in physical activity within a 24-hour day context into account [56,57]. Further research could guide forms of support that health professionals may offer to persons with arthritis using a wearable who struggle to freely direct their own choices about physical activity in their everyday self-management. Without this support, using a wearable in everyday self-management may be experienced as a nagging or autonomy-undermining influence by some persons with arthritis, adding to a struggle to feel in control of their choices to be more active. Evidence exists to indicate that feeling this sense of control in self-management is at least one of the mechanisms responsible for improvements in health behaviors and health status [58-60].

Secondly, our findings build on previous research indicating that persons with arthritis experienced an enhanced awareness of their activity levels when using wearable technology [25,61]. The insights emphasized how a heightened awareness impacted participants’ emotions positively and negatively. Some participants experienced feelings of accomplishment when their wearable data illustrated that they had reached their physical activity goal, which often fueled their motivation to do more. One participant, however, experienced negative thoughts on days that his wearable data indicated he had not reached his physical activity goal. Mercer et al [25] also found that persons with chronic illness (including arthritis) experienced negative feelings in using a wearable during a 15-day research period as they were concerned they were not sufficiently active. From a relational ethics perspective, these experiences speak to a theme of embodiment, emphasizing the importance of complex emotions or feelings in a commitment to ethical action in one’s relationship with oneself and others [34,62]. It remains unknown how others with arthritis may be emotionally impacted if using a wearable in a “real-world” context, outside of research participation. Our findings, therefore, raise questions about how the use of a wearable device within a counseling program may impact the emotional wellbeing of individuals, particularly as they evaluate their capacity and progress in managing their health.

Thirdly, findings indicate how participants experienced issues of accountability and trust differently when reviewing their wearable data with the study PT. Some participants were motivated by a sense of accountability to “keep my promise” to meet the activity goal agreed with the study PT. For some of these participants, wearable data served as “some form of proof” that the study PT could trust to “gauge” whether they had met this goal. One participant, however, expressed how his wearable data was not to be trusted at times and recalled reaching a mutually satisfactory interpretation of this data as inaccurate with the study PT. These different experiences illustrate how sharing wearable data helped build mutual trust and engagement between participants and the study PT. At the same time, this finding also indicates how sharing wearable data threatened to undermine mutual trust and engagement within this relationship.

The fundamental role of building trust in interpersonal relationships in health care has been emphasized elsewhere through a relational ethics lens [62-64]. A relational ethics lens can also be explored here through the relational theme of engagement. Genuine engagement is understood by Bergum [34] to be “located in the shared moment when people have found a way to look at something together, freely accepting or declining the interpretation that each other offers, until they reach a meaning they both affirm.” Our findings support the general opinion previously expressed by persons with OA that sharing their wearable data may enhance their communication with health professionals in everyday self-management under specific conditions (eg, if there was a good rapport already established in the relationship or if a health professional would welcome the wearable data being shared) [20]. They also raise questions about the sense of accountability experienced when sharing wearable data with a health professional, in terms of how far this may be a burden to persons with arthritis in their everyday self-management [65,66]. Further research is therefore needed to gain a better understanding of the relational conditions in which persons with arthritis may experience issues of accountability, trust, and engagement positively or negatively when sharing wearable data with their health professionals in everyday self-management.

### Limitations

There were limitations to this study. As a secondary analysis, the data were not created with the relational ethics lens in mind, and therefore potentially important experiences might not be fully elicited in the interviews. Nonetheless, transcripts were purposively selected to offer sufficient variation in participants’ experiences relevant to our objective. A phenomenographic approach also allowed us to identify overarching meanings that crossed transcripts and were implicitly presented by the collective group. To better examine the transferability of findings, further research is needed to explore the experiences of a more diverse sample using a wearable in the context of their everyday self-management of arthritis outside of research participation. It may be that persons of diverse genders or cultural backgrounds, for example, encounter different experiences. Our subsample, however, is varied and represents a typical OA group in terms of age and sex.

### Conclusions

Our findings provide insight into different ways in which persons with OA experienced their use of a wearable during participation in a physical activity counseling intervention study positively or negatively. Drawing on a relational ethics lens, we identified how issues of relational autonomy, embodiment, accountability, trust, and genuine engagement were present in these experiences. These issues have implications for learning how to develop and implement wearable-enabled physical activity programs to support arthritis self-management in ways that seriously factor in ethical considerations. We present these salient ethical issues for further discussion and to guide future empirical investigation of the use of wearables in arthritis self-management.

## Appendix

Multimedia Appendix 1 Supplementary supporting quotes.

## Abbreviations

MVPA: moderate-to-vigorous physical activity
OA: osteoarthritis
PT: physiotherapist
RCT: randomized controlled trial
