# Supplementary material for: Experiences of Wearable Technology by Persons with Knee Osteoarthritis Participating in a Physical Activity Counseling Intervention: Qualitative Study Using a Relational Ethics Lens
Source: JMIR Mhealth Uhealth. 2021 Nov 12;9(11):e30332. doi: 10.2196/30332 (PMC8663466; doi:10.2196/30332)
Supplement: Multimedia Appendix 1 [file mhealth_v9i11e30332_app1.pdf]

Appendix I: Supplementary Supporting Quotes

Supporting Quotes
**Category 1: Making choices about physical activity with or without a wearable**<br><br>**Biker:** maybe it's a motivator to kind of go… "I've done my 9000 steps. I haven't done 5 miles so maybe I should try and actually go out for a walk" but again if I've been doing a lot of riding then I'll go, "Ah who cares." I haven't done my 5 miles in terms of steps but I've done a lot of miles in terms of bike riding… this has definitely increased my walking and I think maybe my knees are stronger than they were because of the walking and I'm not sure if there's less pain or not… then some days I don't care. Some days I'll go, "Oh I've done 8000. That's enough for today. I've had it," and so that's fine too but just quite often I find that it is a motivator.<br><br>**Marco:** The Fitbit has really encouraged me to walk more… you wanna make sure you get 10,000 steps… prior to being in the study or prior to wearing the Fitbit, I'd have come home and not done too much in the evening. But it made me come home and go for that extra walk, either before or after dinner to get up to whatever my goal was for the day, if it was 10,000 steps.<br><br>**Darius:** So now I'm aware of that so I'm kind of keeping in mind that uh if I, I stay too long I, I walk around and then stand up, do some stretches and things like that… I'm more alert on the daily uh activities. It wouldn't change my routine. I still keep on what I've been doing. But the habits I changed a bit because sometimes if I think I need to uh for example my wife will forget to buy something from the supermarket, instead of driving now I walk. So that, that's the change that I did after joining the research.<br><br>**Logan Kale:** when I would be close to my steps, if I would see…you just tap it and then you'll know if you're close or not and I would just make that extra effort to meet that mark. Like instead of driving to work, I would walk to work. On most days it was easy… The other days I'm just in too much pain. I'm like, "I'm not walking home."<br><br>**Zed:** I don't know if I would continue wearing this after or not because once I find I'm in a, in a structured routine that I'm doing it not for the sake of the Fitbit but I'm just doing it because it's routine… there was a little bit of determination in the beginning that I go oh well, I haven't reached my 10,000, I'd better get on the treadmill, or find something to do or you know so I, that's what I would do but I find that wanes over time… it did start me going, for sure, and then it became a routine. So I think, yeah, I think it started me going but like I said, as time goes on, it's just starting to wane…<br><br>**Hayley:** I'll just say here that this particular Fitbit had very little impact on how I…to motivate me to do exercise for the day… I just didn't find it very useful… you know the choices that I made and the things that I did were made because I decided them, not because the Fitbit had any influence on me at all and so I don't think it changed in that regard.<br><br>**Yoda:** I feel bad saying this because it really shows perhaps an inherent bad attitude but I think I wasn't so invested that I absolutely had to do come hell or high water these steps so I'd be like, "Yeah, I just didn't walk very much today." Um very occasionally I'd go for a walk after everyone was asleep but I generally like to wind down after my kids go to sleep because we all get up very early… I think that goes back to my intrinsic devaluing of the importance of how many steps I do in a day so for me it didn't really bother me because I wasn't absolutely tied to doing a certain amount of steps in a day…
**Category 2: Emotional dimensions of adding awareness about physical activity**<br><br>**Olivia**: Fitbit gave me a way to measure and get the feedback, like instant gratification… It makes me aware. It brings it to a consciousness so it's a good thing to get out… So I don't do a lot but relative to me, it's probably a hundred percent improvement. So that's good… I could see improvement… You know, so it's not an effort. Effort will be getting to 7000. I just…I'd like to be that new normal of at least 5000.

Marco: it was very useful in that I had no idea how many steps I took in a day… really when we started out the goalsetting, idea, I had no idea what to expect so I continued to exceed whatever goals that we set and that was, that's kind of fun. It's very, very motivating, you know. So yeah, I'm, I'm a big believer in the Fitbit. I'll continue to wear it… I've really enjoyed it…
